# Preparation, structure, and reactivity of bicyclic benziodazole: a new hypervalent iodine heterocycle

**DOI:** 10.3762/bjoc.14.87

**Published:** 2018-05-08

**Authors:** Akira Yoshimura, Michael T Shea, Cody L Makitalo, Melissa E Jarvi, Gregory T Rohde, Akio Saito, Mekhman S Yusubov, Viktor V Zhdankin

**Affiliations:** 1 The Tomsk Polytechnic University, 634050 Tomsk, Russia; 2 Department of Chemistry and Biochemistry, University of Minnesota, Duluth, MN 55812, USA; 3 Marshall School, Duluth, MN 55811, USA,; 4 Division of Applied Chemistry, Institute of Engineering, Tokyo University of Agriculture and Technology, 2-24-16 Naka-cho, Koganei, Tokyo 184-8588, Japan

**Keywords:** benziodazole, biheterocycles, hypervalent iodine, iodine, oxidatively assisted esterification

## Abstract

A new bicyclic organohypervalent iodine heterocycle derivative of benziodazole was prepared by oxidation of 2-iodo-*N,N’-*diisopropylisophthalamide with *m*-chloroperoxybenzoic acid under mild conditions. Single crystal X-ray crystallography of this compound revealed a five-membered bis-heterocyclic structure with two covalent bonds between the iodine atom and the nitrogen atoms. This novel benziodazole is a very stable compound with good solubility in common organic solvents. This compound can be used as an efficient reagent for oxidatively assisted coupling of carboxylic acids with alcohols or amines to afford the corresponding esters or amides in moderate yields.

## Introduction

**Scheme 1:**
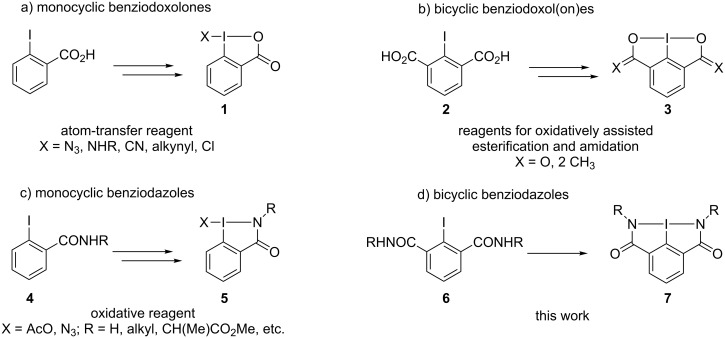
Representative examples of benziodoxoles and benziodazoles.

In recent years, the interest in heterocyclic organohypervalent iodine compounds has experienced an unprecedented growth [1–6]. A variety of new hypervalent iodine heterocycles have been prepared, and numerous reactions employing these compounds as reagents for organic synthesis have been reported. The benziodoxole-based five-membered iodine heterocycles represent a particularly important class of hypervalent iodine(III) reagents. Substituted benziodoxoles **1** (Scheme 1a) are commonly employed as efficient electrophilic atom-transfer reagents useful for conversion of various organic substrates to the corresponding products of azidation [7–11], amination [12–13], cyanation [14–17], alkynylation [18–20], or chlorination [21–22]. Recently, Zhang and co-workers reported the preparation of several bicyclic benziodoxoles **3** starting from 2-iodoisophthalic acid (**2**, Scheme 1b). These bicyclic benziodoxoles **3** can be used as efficient coupling reagents for the direct condensation reaction between carboxylic acids and alcohols or amines to provide esters, macrocyclic lactones, or amides and peptides [23–25].

Numerous examples of five-membered hypervalent iodine(III) heterocycles containing other than oxygen heteroatoms, such as sulfur [26], boron [27–28], phosphorous [29], or nitrogen [30–32], have been synthesized and characterized by X-ray crystallography. In particular, several nitrogen containing heterocyclic iodine(III) compounds **5**, benziodazoles, have been reported by Gougoutas [31], Balthazor [32], and our group [33–35] (Scheme 1c). X-ray structural studies of these benziodazoles confirmed the presence of covalent bonding between iodine and nitrogen atoms in the heterocyclic ring. Benziodazoles **5** are usually prepared by the treatment of 2-iodobenzamide derivatives **4** with appropriate oxidants under mild conditions [31–35]. Derivatives of benziodazole can be used as reagents for various oxidative functionalizations of organic substrates [33,36]. For example, azidobenziodazole was used as an efficient azidation reagent with a reactivity similar to azidobenziodoxoles [33]. Recently, the Wang group reported a rhenium catalyst-mediated oxidative dehydrogenative olefination of a C(sp^3^)–H bond using acetoxybenziodazole reagents [36]. To the best of our knowledge, all known benziodazoles have a mono-heterocyclic structure, and bi-heterocyclic benziodazole derivatives similar to the bicyclic benziodoxole **3** have never been reported. In this paper, we report the synthesis, structural characterization, and reactivity of a novel bicyclic benziodazole derivative **7** (Scheme 1d).

## Results and Discussion

**Scheme 2:**
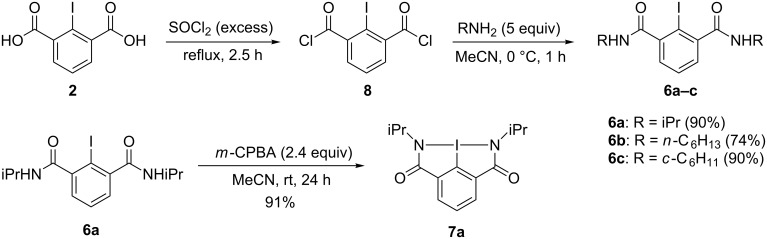
Preparation of bicyclic benziodazole **7a**.

An obvious approach to the preparation of bicyclic benziodazoles **7** involves the oxidation of the corresponding 2-iodo-*N,N’-*dialkylisophthalamides **6** (Scheme 1). We have synthesized the precursors **6** in two simple steps starting from commercially available 2-iodoisophthalic acid (**2**). Firstly, 2-iodoisophthalic acid (**2**) was converted to the corresponding acyl chloride **8** by treatment with thionyl chloride (Scheme 2). In the second step, acyl chloride **8** reacted with appropriate alkylamines to give the corresponding 2-iodo-*N,N’-*dialkylisophthalamides **6** in good yields. The oxidation of 2-iodo-*N,N’-*diisopropylisophthalamide (**6a**) with *m*-chloroperoxybenzoic acid (*m*CPBA) under mild conditions afforded the desired bicyclic benziodazole **7a** in good yield. Unfortunately, we could not obtain the corresponding pure benziodazole derivatives **7** by the oxidation of precursors **6b** or **6c** under similar conditions. According to NMR spectra of the reaction mixture of **6b** or **6c**, the desired products **7b** or **7c** were observed in the reaction as a complex mixture with other compounds. Bicyclic benziodazole **7a** is a thermally stable, white, microcrystalline compound that can be stored in a refrigerator for several weeks. Solutions of **7a** in CDCl_3_ or CD_3_CN did not show any decomposition even after storage for over one month at room temperature.

**Figure 1:**
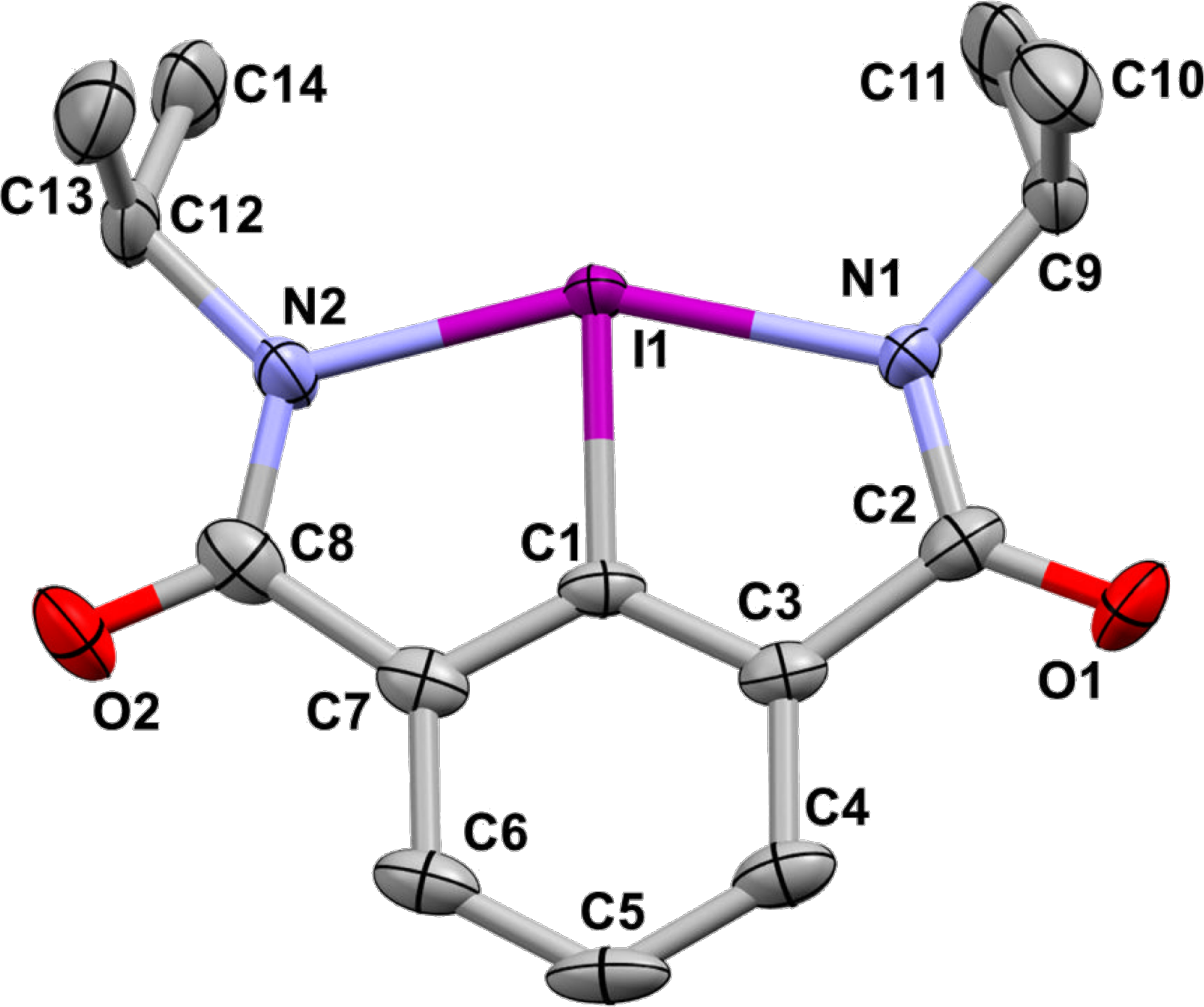
X-ray crystal structure of compound **7a**. Ellipsoids are drawn to the 50% probability level. Selected bond lengths and angles: I(1)–C(1) 2.040 (4) Å; I(1)–N(1) 2.184 (4) Å; I(1)–N(1) 2.177 (4) Å; N(1)–I(1)–C(1) 76.89 (18)°; N(2)–I(1)–C(1) 77.02 (18)°; N(1)–I(1)–N(2) 153.90 (15)°.

The solid state structure of compound **7a** was characterized by X-ray crystallography. A single crystal X-ray diffraction of **7a** confirmed the bicyclic benziodazole structure with two covalent bonds between the iodine atom and the nitrogen atoms I(1)–N(1) = 2.184 (4) Å, I(1)–N(2) = 2.177 (4) Å (Figure 1). These bond lengths are similar to previously reported benziodazole structures [29–32]. According to X-ray crystallography data, structure **7a** has a distorted T-shaped geometry with an N(1)–I(1)–N(2) angle of 153.90 (15)°. Compared to other reported bicyclic hypervalent iodine compounds [23,25,37], this is the most bent structure at the N(1)–I(1)–N(2) angle. An additional relatively weak intermolecular coordination between the iodine atom and the oxygen atom of a neighboring molecule (I(1)**···**O’(1) = 3.107 (3) Å) results in the overall pseudo-square planar geometry at the iodine center.

**Scheme 3:**
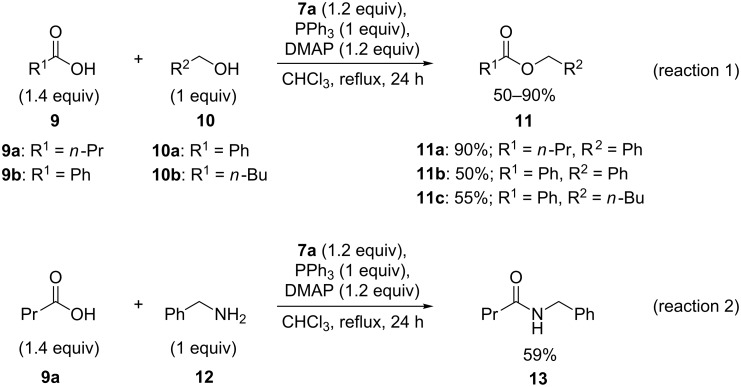
Benziodadiazole **7a** mediated oxidatively assisted esterification and amidation reactions.

Similar to the iodosodilactone reagents [23–25], the bicyclic benziodazole **7a** could be expected to be a useful reagent for oxidatively assisted coupling reactions. Previously, Zhang and co-workers reported the reactions of carboxylic acids with alcohols or amines in the presence of stoichiometric amounts of iodosodilactones **3** forming the corresponding esters or amides in moderate to good yields via an oxidatively assisted coupling reaction [23–25]. We have investigated the analogous oxidatively assisted coupling reaction of carboxylic acids **9** with alcohols **10** or amine **12** using benziodazole **7a** under similar conditions (Scheme 3). The reaction of butyric acid (**9a**) with benzyl alcohol (**10a**) using benziodiazole **7a** in the presence of triphenylphosphine and *N*,*N*-dimethyl-4-aminopyridine (DMAP) in chloroform solution under reflux conditions afforded the desired product **11a** in good yield. As expected, the reactions of benzoic acid (**9b**) with benzyl alcohol (**10a**) or 1-pentanol (**10b**) under the same conditions gave the corresponding esters **11b** or **11c** in moderate yields (Scheme 3, reaction 1). The analogous reaction of butyric acid (**9a**) with benzylamine (**12**) and benziodazole **7a** under similar conditions produced the expected amide **13** in moderate yield (Scheme 3, reaction 2). Compared to the iodosodilactone reagents **3** [23–25], benziodazole **7a** showed a comparable or better reactivity. In contrast to iodosodilactone, benziodazole **7a** has excellent solubility in chloroform allowing reactions in solution under homogeneous conditions. Similar to the reactions of iodosodilactone **3**, Ph_3_P=O and amide **6a** were observed as the byproducts in these reactions (Scheme 3), which is in agreement with the previously proposed mechanism of oxidatively assisted esterification or amidation [23,38].

## Conclusion

In summary, we have prepared the new bicyclic benziodazole **7a** by the oxidation of 2-iodo-*N,N’-*diisopropylisophthalamide (**6a**) with *m*-CPBA. The solid structure of **7a** was established by X-ray crystallography. According to the X-ray data, this compound has a bis-heterocyclic structure with two covalent iodine–nitrogen bonds and distorted T-shape geometry at the hypervalent iodine center. This novel bicyclic benziodazole can be used as an efficient reagent for oxidatively assisted coupling of carboxylic acids with alcohols or amines to afford the corresponding esters or amides in moderate to good yields.

## Supporting Information

File 1 Experimental section.

File 2 X-ray structure of **7a**.
