# Supplementary material for: Preparation, structure, and reactivity of bicyclic benziodazole: a new hypervalent iodine heterocycle
Source: Beilstein J Org Chem. 2018 May 8;14:1016–20. doi: 10.3762/bjoc.14.87 (PMC6009394; doi:10.3762/bjoc.14.87)

Supporting Information

for

Preparation, structure, and reactivity of bicyclic benziodazole: a new hypervalent iodine heterocycle

Akira Yoshimura*[1,2], Michael T. Shea[2], Cody L. Makitalo[2], Melissa E. Jarvi[2], Gregory T. Rohde[3], Akio Saito[4], Mekhman S. Yusubov[1] and Viktor V. Zhdankin*[1,2]

Address: [1]The Tomsk Polytechnic University, 634050 Tomsk, Russia, [2]Department of Chemistry and Biochemistry, University of Minnesota, Duluth, MN 55812, USA, [3]Marshall School, Duluth, MN 55811, USA, and [4]Division of Applied Chemistry, Institute of Engineering, Tokyo University of Agriculture and Technology, 2-24-16 Naka-cho, Koganei, Tokyo 184-8588, Japan

Email: Akira Yoshimura* - ayoshimu@d.umn.edu, Viktor V. Zhdankin* - vzhdanki@d.umn.edu

* Corresponding author

Experimental section

Table of Contents: pp

1. General experimental remarks S2

2. Preparation of 2-iodo-*N*,*N*'-diisopropylisophthalamide (**6a**) S2

3. Preparation of *N*,*N*'-diisopropylbenziodazole (**7a**) S3

4. General procedure for oxidatively assisted coupling reactions of carboxylic acids with alcohols or amine S4

5. References S6

6. Spectra of products S7

General experimental remarks

All reactions were performed under dry argon atmosphere with flame-dried glassware. All commercial reagents were ACS reagent grade and used without further purification. Dichloromethane was distilled from CaH_2 immediately prior to use. Diethyl ether was distilled from Na/benzophenone. All commercial reagents were ACS reagent grade and used without further purification. Melting points were determined in an open capillary tube with a Mel-temp II melting point apparatus. Infrared spectra were recorded as a KBr pellet on a Perkin-Elmer 1600 series FT-IR spectrophotometer. NMR spectra were recorded on a Varian Inova 500 or 300 MHz NMR spectrometer at 500 or 300 MHz (^{1}H NMR) and 125 MHz or 75 MHz (^{13}C NMR). Chemical shifts are reported in parts per million (ppm). ^{1}H and ^{13}C chemical shifts are referenced relative to tetramethylsilane.

Preparation 2-iodo-*N*,*N*'-diisopropylisophthalamide (6a)

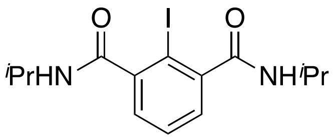

A solution of 2-iodoisophthalic acid (**2**, 580 mg, 0.200 mmol) in thionyl chloride (2.0 mL) was refluxed for 2.5 h. After that, the solvent was removed under reduced pressure to give the crude solid acyl chloride **8**. Then diisopropylamine (590 mg, 1.0 mmol) was added at 0 °C to a stirred mixture of crude acyl chloride **8** (660 mg, 0.200 mmol) in MeCN (2.0 mL). The reaction mixture was stirred at 0 °C for 1 h. After completion of reaction, the solvent was removed under reduced pressure to give a solid residue which was recrystallized from dichloromethane/hexane solution to give pure amide **6a**. Yield 740 mg (90%, 2 steps), isolated as a white-solid, mp 259.5-260.4 °C; IR (neat) cm^{-1}: 3277, 3067, 2970, 2936, 2876, 1645, 1539; ^{1}H NMR (500 MHz, $CDCl_3$): δ 7.35 (t, *J* = 7.1 Hz, 1H), 7.27 (d, *J* = 7.1 Hz, 2H), 5.79 (d, *J* = 7.0 Hz, 2H), 4.34-4.21 (m, 2H), 1.29 (d, *J* = 7.0 Hz, 12H); ^{13}C NMR (75 MHz, $CDCl_3$): δ 168.8, 144.5, 128.5, 128.2, 90.7, 42.3, 22.6; HRMS (APCI-positive): calcd for $C_{14}H_{20}IN_2O_2$ ($[M+H])^+$: 375.0569, found: 375.0574.

Preparation of *N*,*N*'-diisopropylbenziodazole (7a)

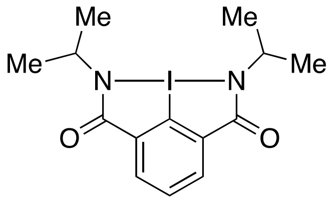

Amide **6a** (670 mg, 0.20 mmol) was added to a solution of *m*-CPBA (830.0 mg, 0.480 mmol) in MeCN (3.0 mL). The reaction was stirred at room temperature for 24 h. After completion of reaction, the solvent was removed under reduced pressure to give solid residue. Then diethyl ether was added to solid residue to prepare the suspended solution, which was filtered, washed with diethyl ether several times, and dried in vacuum to give product **7a**. Yield 680 mg (91%), isolated as a white solid, mp 143.8 °C (decomp.); IR (CH_2Cl_2) cm^{-1}: 3071, 3039, 2965, 2929, 2872, 1626, 1584; ^{1}H NMR (500 MHz, $CDCl_3$): δ 8.32 (d, *J* = 7.1 Hz, 2H), 7.90 (t, *J* = 7.1 Hz, 1H), 4.41 (sept, *J* = 6.8 Hz, 2H), 1.41 (d, *J* = 6.8 Hz, 12H); ^{13}C NMR (75 MHz, $CDCl_3$): δ 162.8, 132.7, 132.2, 131.2, 113.1, 46.3, 24.5; HRMS (APCI): calcd for $C_{14}H_{18}IN_2O_2$ ([M+H])$^+$: 373.0413, found: 373.0398.

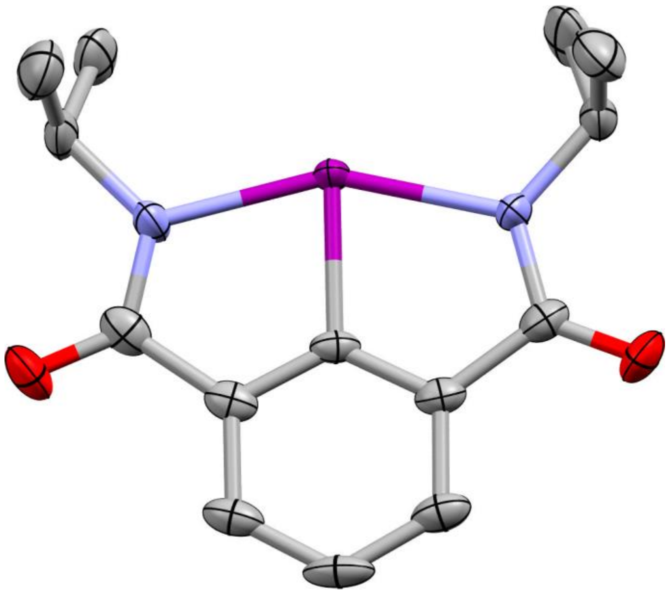

Figure S1: X-ray structure of *N*,*N*'-diisopropylbenziodazole (**7a**).

Single crystals of product **7a** suitable for X-ray crystallographic analysis were obtained by slow crystallization from dichloromethane solution. X-ray diffraction data for **7a** were collected on Rigaku RAPID II Image Plate system using graphite-monochromated Mo Kα radiation (λ = 0.71073 Å) at 173 K. The structure was solved by DIRDIF v2008.3[1] and refined using SHELXL-2014/7[2]. Crystal data for **7a** $C_{14}H_{17}IN_2O_2$, orthorhombic, space group $Pna2_1$, a = 17.695(2) Å, b = 9.0710(10) Å, c = 9.1760(10) Å, α = 90 °, β = 90 °, γ = 90 °, V = 1472.9(3) $Å^3$, Z = 4, 12610 reflections measured, 3294 unique reflections, 2580 I > 2/s(I), 176 parameters, 1 restraints; Flack Parameter 0.04(2); GooF = 1.038, final R1 = 0.0223, Rw (all) = 0.0510. CCDC 1821160.

General procedure for oxidatively assisted coupling reactions of carboxylic acids with alcohols or amine

A mixture of **7a** (67 mg, 0.180 mmol) and DMAP (22 mg, 0.180 mmol) in $CHCl_3$ (5.0 mL) was stirred at reflux for 1 h. Then, carboxylic acid **9** (0.210 mmol), PPh_3 (39 mg, 0.150 mmol) and alcohol **10** (0.150 mmol) or amine **12** (0.150 mmol) were added to the solution. The reaction mixture was stirred at reflux for 24 h. After reaction, saturated aqueous $NaHCO_3$ (5 mL) was added and the mixture was extracted with dichloromethane. The organic layer was dried over anhydrous Na_2SO_4 and concentrated under reduced pressure. Purification by preparative TLC (hexane/ethyl acetate = 1:1) afforded the analytically pure **11** or **13**.

Benzyl butyrate (**11a**)[3]

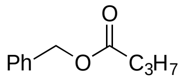

Reaction of butyric acid (**9a**, 19 mg, 0.210 mmol) and benzyl alcohol (**10a**, 16 mg, 0.150 mmol) according to the general procedure afforded 24 mg (90%) of product **11a**, isolated as a colorless oil; IR (neat) cm^{-1}: 3067, 3036, 2966, 2935, 2877, 1738, 1258, 1172; ^1H NMR (300 MHz, $CDCl_3$): δ 7.40-7.29 (m, 5H), 5.12 (s, 2H), 2.34 (t, J = 7.4 Hz, 2H), 1.76-1.60 (m, 2H), 0.95 (t, J = 7.2 Hz, 3H); ^{13}C NMR (75 MHz, $CDCl_3$): δ 173.5, 136.2, 128.5, 128.2, 66.1, 36.2, 18.5, 13.7.

Benzyl benzoate (**11b**)[4]

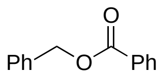

Reaction of benzoic acid (**9b**, 26 mg, 0.210 mmol) and benzyl alcohol (**10a**, 16 mg, 0.150 mmol) according to the general procedure afforded 16 mg (50%) of product **11b**, isolated as a colorless oil; IR (neat) cm^{-1}: 3090, 3062, 3032, 2954, 1720, 1602, 1452, 1272, 1110, 712; ^{1}H NMR (500 MHz, $CDCl_3$): δ 8.08 (d, *J* = 6.5 Hz, 2H), 7.55 (t, *J* = 7.5 Hz, 1H), 7.47-7.31 (m, 7H), 5.37 (s, 2H); ^{13}C NMR (75 MHz, $CDCl_3$): δ 166.4, 136.1, 133.0, 130.2, 129.7, 128.6, 128.4, 128.2, 128.2, 66.7.

Pentyl benzoate (**11c**)[5]

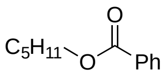

Reaction of benzoic acid (**9b**, 26 mg, 0.210 mmol) and 1-pentanol (**10b,** 13 mg, 0.150 mmol) according to the general procedure afforded 16 mg (55%) of product **11c**, isolated as a colorless oil; IR (neat) cm^{-1}: 3070, 2959, 2933, 2783, 2862, 1722, 1603, 1452, 1274, 1109, 710; ^{1}H NMR (500 MHz, $CDCl_3$): δ 8.08 (d, *J* = 6.5 Hz, 2H), 7.55 (t, *J* = 7.5 Hz, 1H), 7.47-7.31 (m, 7H), 5.37 (s, 2H); ^{13}C NMR (75 MHz, $CDCl_3$): δ 166.9, 134.0, 130.7, 129.5, 128.6, 65.3, 28.6, 28.4, 22.5, 15.0.

Benzylbutyramide (**13**)[3]

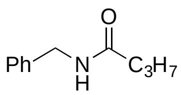

Reaction of butyric acid (**9a**, 19 mg, 0.210 mmol) and benzylamine (**12**, 16 mg, 0.150 mmol) according to the general procedure afforded 16 mg (59%) of product **13**, isolated as a colorless oil; IR (neat) cm^{-1}: 3294, 3087, 3032, 2961, 2931, 2873, 1634, 1553, 1454; ^{1}H NMR (500 MHz, $CDCl_3$): δ 7.36-7.31 (m, 3H), 7.28 (d, *J* = 6.5 Hz, 2H), 5.66 (brs, 1H), 4.45 (d, *J* = 5.5 Hz, 2H), 2.20 (t, *J* = 7.5 Hz, 2H), 1.75-1.65 (m, 2H), 0.97 (t, *J* = 7.5 Hz, 2H).

References

1. Beurskens, P. T.; Beurskens, G.; de Gelder, R.; Garcia-Granda, S.; Gould, R. O.; Smits, J. M. M. *The DIRDIF2008 program system, Crystallography Laboratory, University of Nijmegen, The Netherlands,* 2008.

2. Sheldrick, G. M. *Acta Crystallogr.* **2015**, *C71* 3-8.

3. Nguyen, T. V.; Bekensir, A. *Org. Lett.* **2014**, *16*, 1720-1723.

4. Doi, R.; Kikushima, K.; Ohashi, M.; Ogoshi, S. *J. Am. Chem. Soc.* **2015**, *137*, 3276-3282.

5. Gowrisankar, S.; Neumann, H.; Beller, M. *Angew. Chem., Int. Ed.* **2011**, *50*, 5139-5143.

^{1}H NMR (500 MHz, $CDCl_3$)

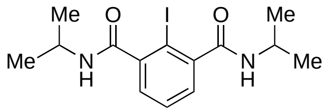

6a

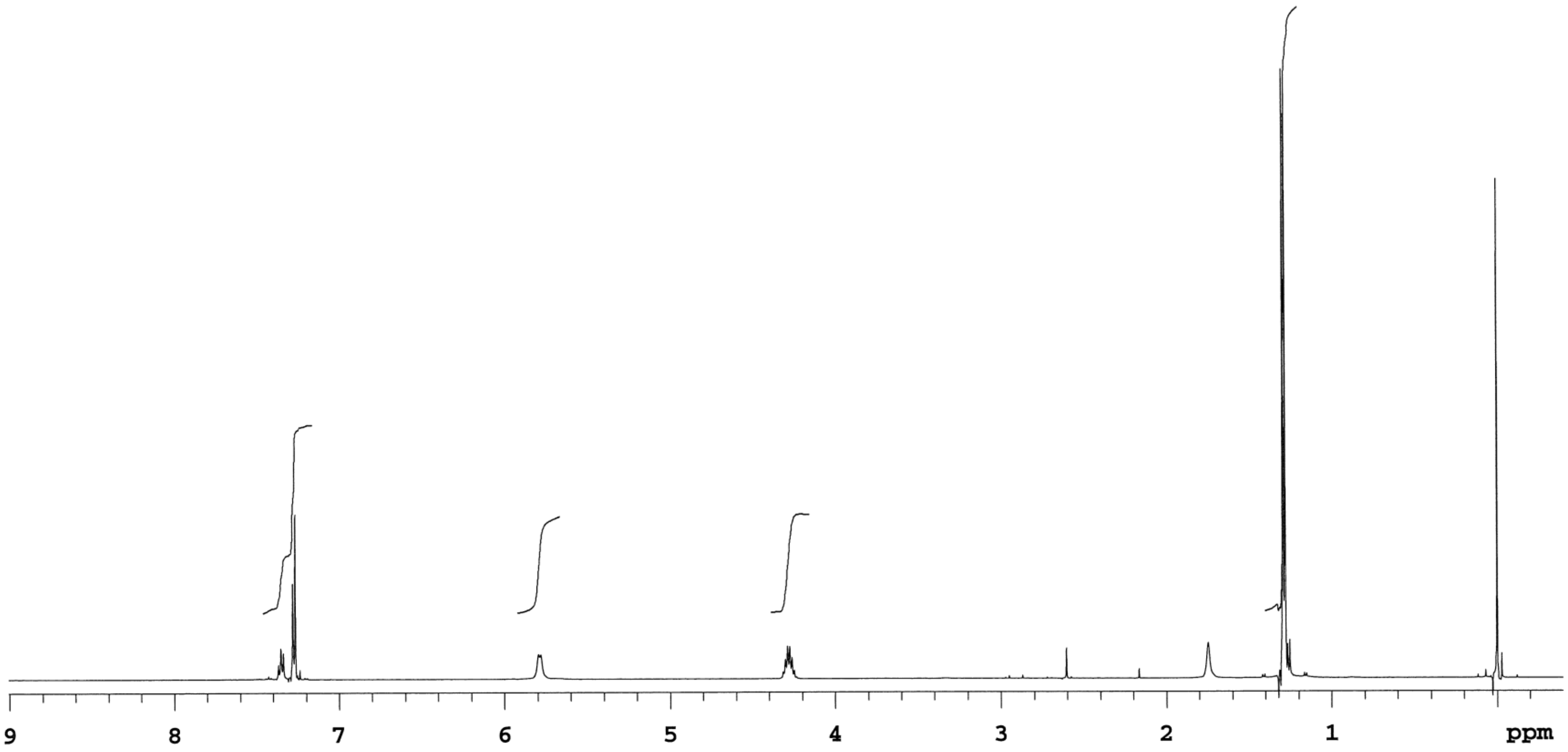

^{13}C NMR (75 MHz, $CDCl_3$)

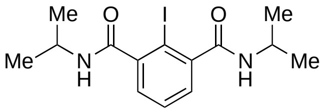

6a

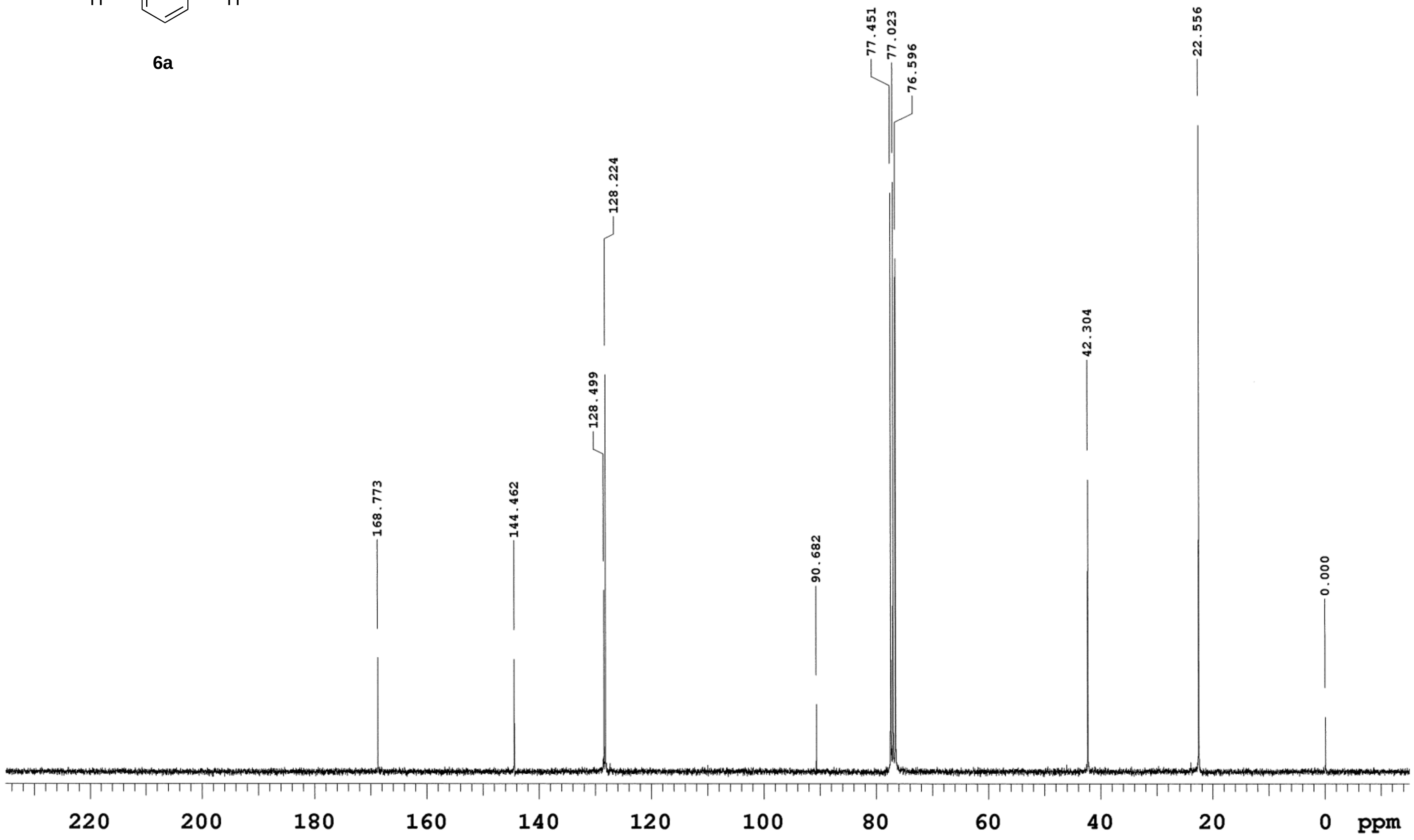

^{1}H NMR (300 MHz, $CDCl_3$)

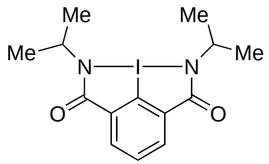

7a

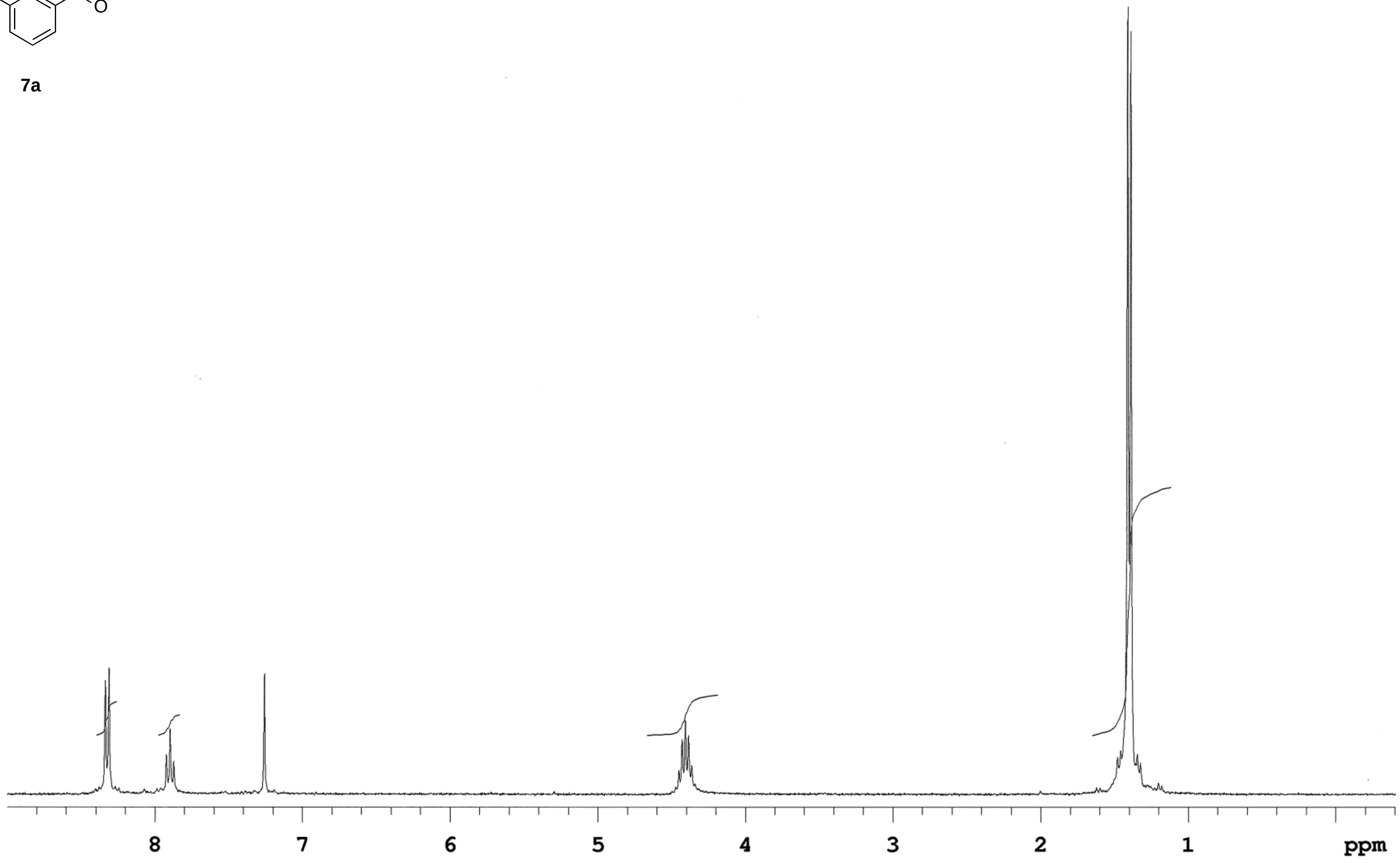

^{13}C NMR (75 MHz, $CDCl_3$)

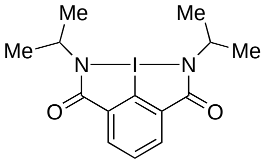

7a

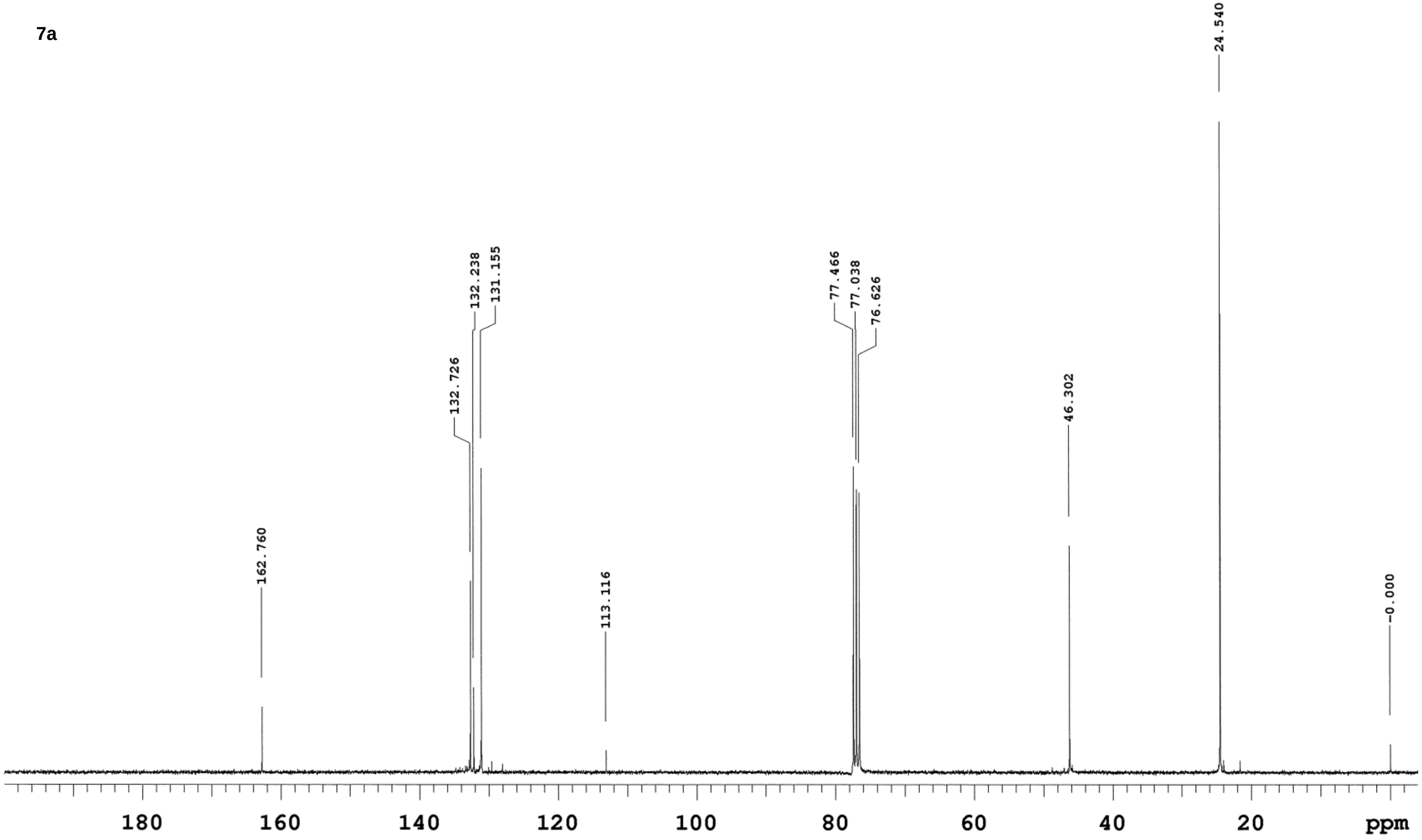

^{1}H NMR (300 MHz, $CDCl_3$)

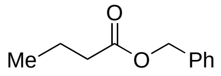

11a

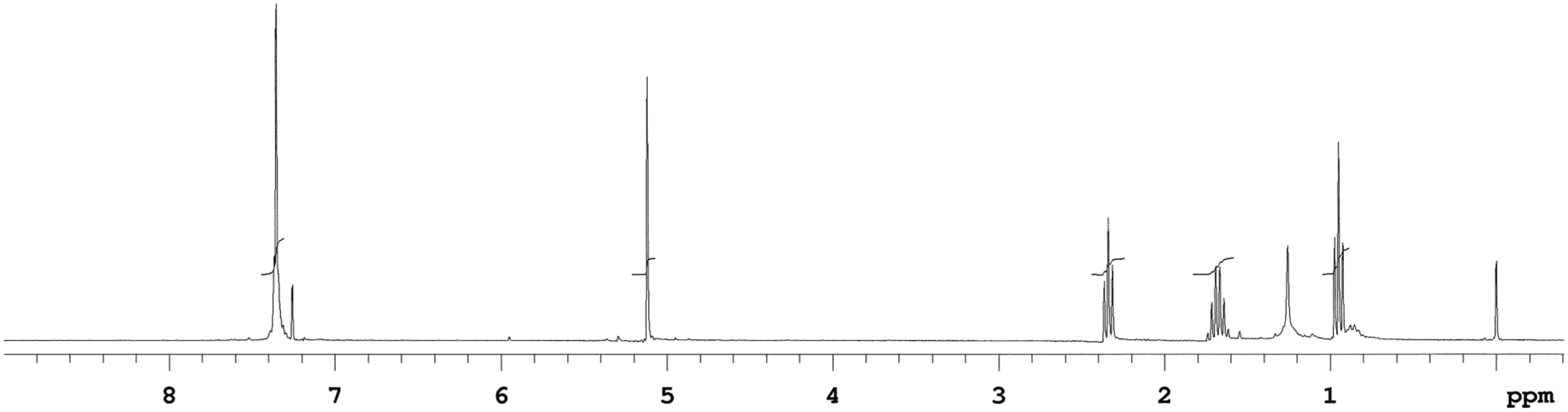

^{1}H NMR (500 MHz, $CDCl_3$)

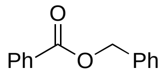

11b

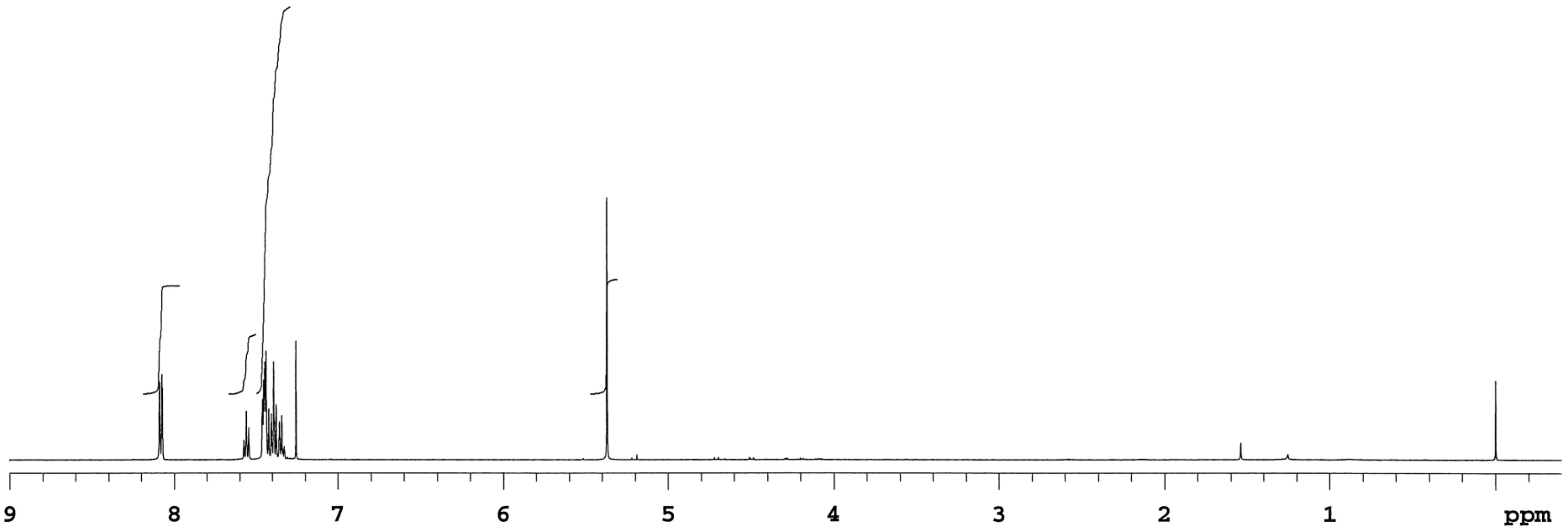

^{1}H NMR (500 MHz, $CDCl_3$)

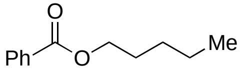

11c

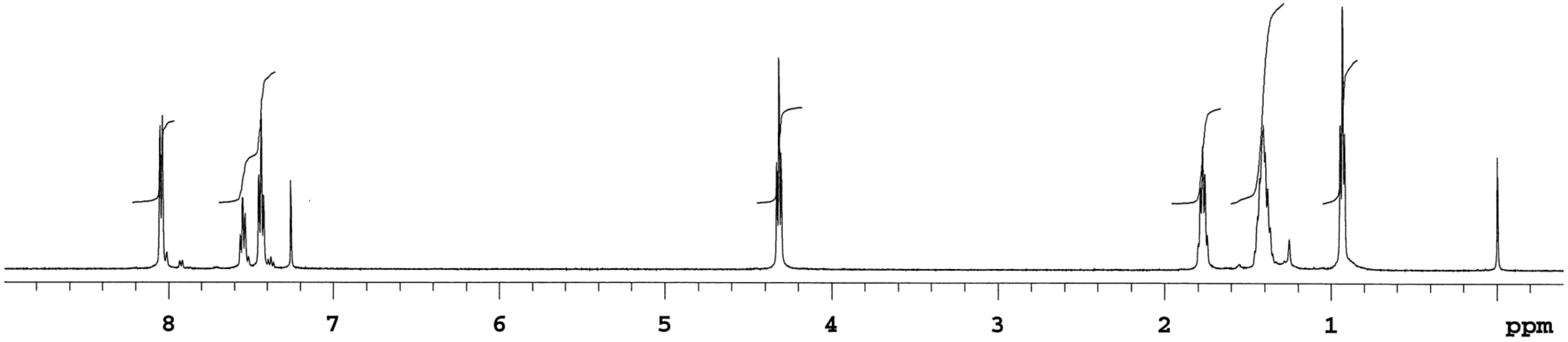

^{1}H NMR (500 MHz, $CDCl_3$)

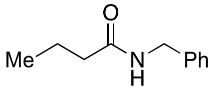

13

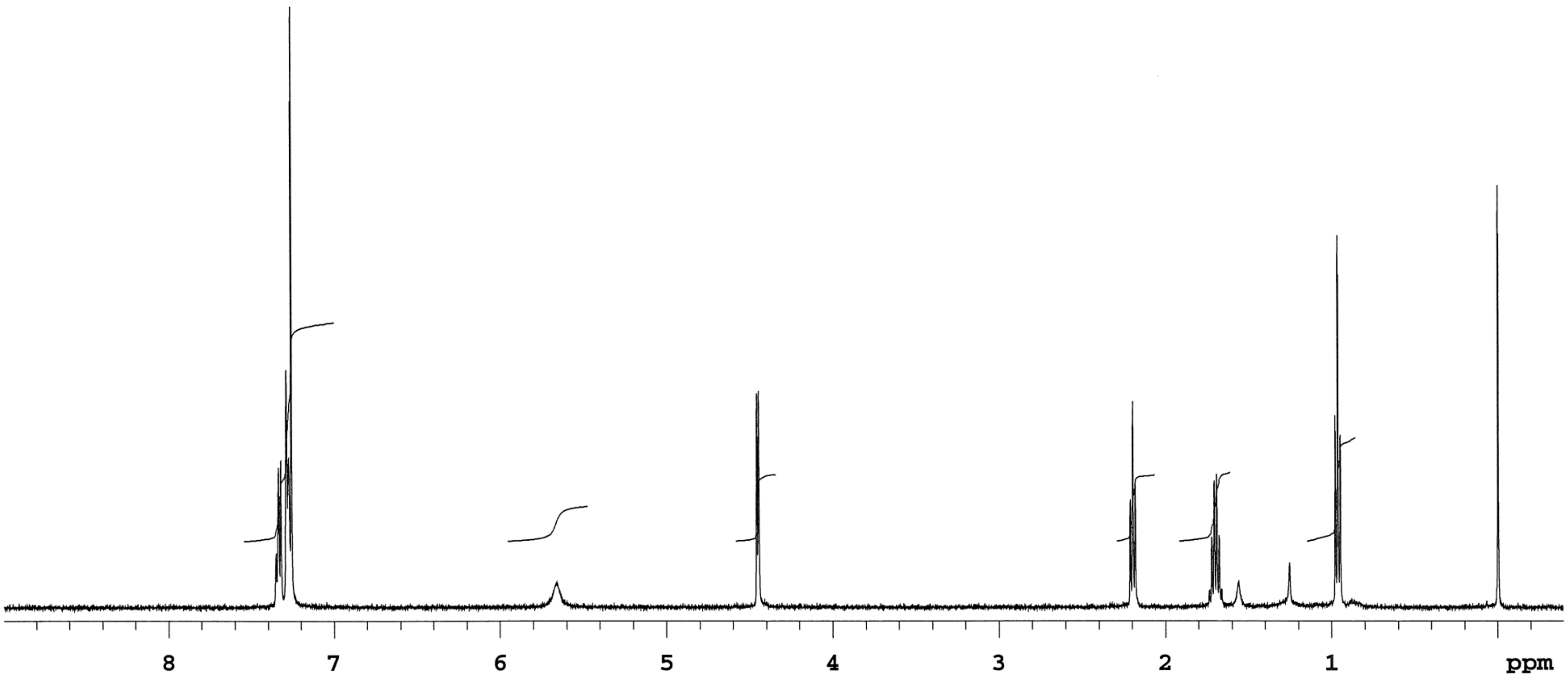

Supplement: File 1 — Experimental section. [file Beilstein_J_Org_Chem-14-1016-s001.pdf]
